# Sound Velocity and Equation of State of Ballistic Gelatin by Brillouin Scattering

**DOI:** 10.3390/ma16031279

**Published:** 2023-02-02

**Authors:** Muhtar Ahart, Russell J. Hemley

**Affiliations:** 1 Department of Physics, University of Illinois Chicago, Chicago, IL 60607, USA; 2 Department of Chemistry, University of Illinois Chicago, Chicago, IL 60607, USA; 3 Department of Earth and Environmental Sciences, University of Illinois Chicago, Chicago, IL 60607, USA

**Keywords:** Brillouin scattering, equation of state, ballistic gelatin, high-pressure

## Abstract

Brillouin scattering spectroscopy with diamond anvil cells was used by measuring the pressure dependence of the sound-relevant polymer material, glass-forming liquid, and H_2_O (water and ice VII) velocities of the material from ambient pressure to 12 GPa at room temperature. Measurements of 20%, 10%, and 4% gelatin solutions were performed. For comparison purposes, we also measured the pressure dependence of the sound velocity of animal tissue up to 10 GPa. We analyzed the Brillouin data using the Tait and Vinet equations of state. We discussed the possible influence of frequency dispersion on bulk modulus at low pressure. We compared the elastic moduli obtained for gelatin to those of several other polymers.

## 1. Introduction

External stimuli, such as temperatures and pressure, have a pronounced influence on the mechanical behavior of polymers. An important biopolymer is gelatin, a yellow-white protein-rich powder extracted by acid or base hydrolysis from collagen, the main matrix material of animal skin, bone, and connective tissue. Gelatin also refers to aqueous solutions of this powder. As an aggregate material, the molecular weight ranges between 17,000 and 300,000 Daltons. Thus, only the average molecular weight can be used to characterize the material [1]. Gelatin is one of the most versatile hydrocolloids and has a wide variety of industrial uses, including food, pharmaceuticals, and photography. Another use of this polymer gel is as the model of the effects of traumatic impacts on animal tissue, i.e., bullet penetration of so-called “ballistic gelatin” [1], for which the elastic behavior and equation of state (EOS) to gigapascal pressures are of interest.

Despite numerous studies of polymer gels [2,3], proteins [4], and aqueous solutions [5,6] as a function of temperature, limited experiments have been performed to examine pressure effects on these materials. Some efforts have been made to investigate the glass transition and EOS of glass-forming liquids under pressure by Brillouin scattering [7,8]. Similar investigations of the EOS and elastic properties of various polymers under pressure have been conducted during the past decade. For example, the EOS and elastic properties of three elastomers, sylgard (a crosslinked polydimethylsiloxane), estane (a segmented polyester-polyurethane copolymer), and VCE (a crosslinked terpolymer poly-ethylene-vinyl acetate-vinyl alcohol), have been studied by high-pressure Brillouin scattering up to 10 GPa [9]; Kel-F 800, a binder for explosives, has been measured up to 85 GPa by Brillouin scattering, and the EOS and pressure-dependent elastic properties were obtained [10,11].

Here we present the results of a detailed study of gelatin water solution in diamond anvil cells (DACs) in conjunction with Brillouin scattering from ambient pressure to 12 GPa. Motivated the implications for traumatic impacts on animal tissue, we also measured the pressure dependence of the sound velocities of several animal tissues, i.e., lamb muscle and brain tissues. The results extend the EOS and provide additional analyses of experimental data presented previously for the material in an unpublished report [12]. Specifically, we assess the elastic parameters and compare related materials that provide insight into the microscopic structure of these materials.

## 2. Experimental Methods

### 2.1. Sample Preparation

**Figure 1:**
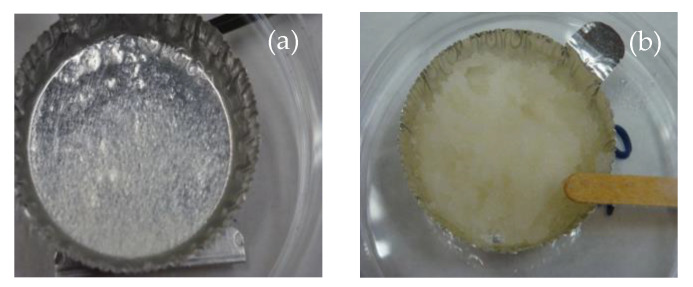
Images of gelatin solutions taken after the gelatin powders were mixed with water; (**a**) 4% solution; (**b**) 20% solution.

**Figure 2:**
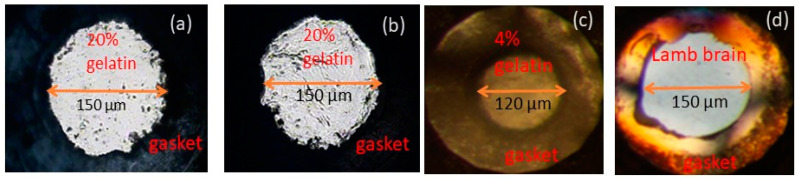
The photographs of gelatin solutions and animal tissue in DACs; (**a**) 20% gelatin solution at 2 GPa, 150 µm gasket hole; (**b**) 20% gelatin solution at 11 GPa, gasket hole is same as (**a**); (**c**) 4% gelatin solution at 1 GPa, 120 µm gasket hole; and (**d**) lamb brain tissue at 2 GPa, 150 µm gasket hole.

The samples of gelatin (20%, 10%, and 4%) were prepared with the methods described in [1]. Images of as-made 4% and 20% gelatin solution are shown in Figure 1. Before loading, gelatin solutions were refrigerated for at least 24 h. Gelatin samples were then loaded into diamond anvil cells (DACs) (Figure 2). The DACs consist of two opposed diamond anvils on the piston and cylinder sides of a cell, with a metal gasket with a hole in its center used as a sample chamber sandwiched between the diamonds [13]. Preindented stainless gaskets with gasket holes of 120–150 µm were used. We used the standard ruby fluorescence method [14] to determine the pressure. We measured the pressure dependencies of velocities of 20%, 10%, and 4% gelatin solutions as well as fresh lamb muscle and brain tissues. These experiments did not involve the use of a separate pressure medium because the materials studied remain soft and serve as a hydrostatic or quasi-hydrostatic medium.

### 2.2. Brillouin Scattering

Brillouin light scattering is the inelastic scattering of an incident optical wave field by thermally excited elastic waves in a material [15,16]. The technique has been widely used in biology [17], chemistry [18], earth science [19], materials science [20], and polymer science [10]. In Brillouin scattering, incident light induces dynamic fluctuations in the strain field to bring about fluctuations in the dielectric constant; these in turn translate into fluctuations in the refractive index due to elasto-optic scattering. The phonons in the material move in thermal equilibrium with very small amplitudes and may be viewed as a moving diffraction grating by an incident light wave. The frequency shift of inelastically scattered light is given by

(1) $$\Delta \nu =\frac{\upsilon }{\lambda }\sqrt{\left(n_{i}^{2}+n_{s}^{2}-2n_{i}n_{s}\cos\theta \right)}$$

where Δν is the Brillouin shift, *λ* is the wavelength of incident light, *n_i_* is the refractive index in the direction of the incident light, *n_s_* is the refractive index in the scattering direction, *υ* is the velocity of acoustic phonons, and *θ* is the scattering angle.

**Figure 3:**
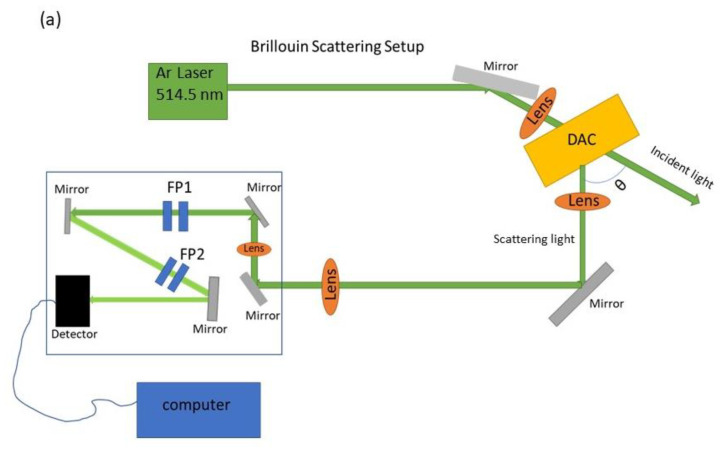

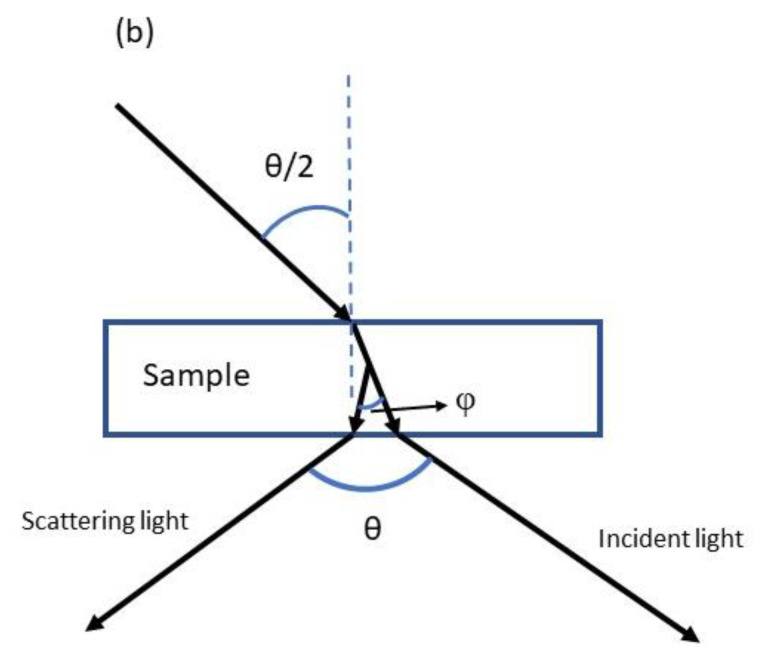
Schematic diagrams of Brillouin scattering setup and the symmetric scattering geometry. (**a**) Incident light is focused on the sample by the first lens, with scattered light collected by second lens, sent to the Fabry–Perot interferometer (FP1 and PP2), and measured by the detector. (**b**) The enlarged sample region inside of a DAC, showing the incident light entering the sample with the angle *θ*/2. By Snell’s law, *n*_0_sin(*θ*/2) = *n*sin(*φ*/2), where *n*_0_ is the refractive index of air and *n* is the refractive index of samples. Therefore, the Brillouin shifts have the following form: $\Delta \nu_{\phi }=\frac{2n\upsilon }{\lambda }\sin(\phi /2)=\frac{2\upsilon }{\lambda }\sin(\theta /2)=\Delta \nu_{\theta }$. The Brillouin shifts are uniquely defined by the scattering angle between the incident and scattering light, as the sound velocity of the sample (assumed to be isotropic) does not depend on the scattering angle.

In the present experiments, light from a single-mode Ar-ion laser was used as the excitation source with an average power of 100 mW. The DAC sample was placed symmetrically with respect to the incoming and collected light such that the difference vector of the incoming and detected light is in the plane of the sample. Scattering angles of 80° and 70° degrees were employed in these experiments (Figure 3). Since samples were placed symmetrically with respect to the incoming and collected light, the frequency shift of the incident light is independent of the refractive index of the samples. Thus, Equation (1) can be reduced to

(2) Δν=(2υ/λ)sin(θ/2)

where *υ* is sound velocity, *λ* = 514.5 nm (laser wavelength), and *θ* is the scattering angle. The scattered light was collected by a lens and analyzed by a 3 + 3 tandem Fabry–Perot interferometer, detected by a photon-counting photomultiplier, and output to a multichannel scalar (Figure 3a). Spectra were collected for 10 to 120 min at each pressure at 295 K. Additional details about the Brillouin scattering system setup can be found in [21,22,23].

## 3. Results and Analysis

### 3.1. Brillouin Scattering

**Figure 4:**
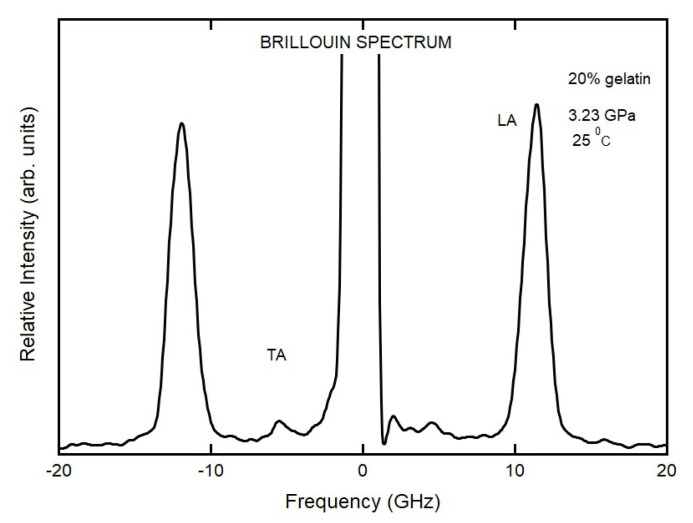
Representative Brillouin spectrum for 20% gelatin solutions. The elastic scattering (Rayleigh) line shown at zero frequency. The Brillouin peaks appear at around 5 and 12.5 GHz for transverse acoustic (TA) and longitudinal acoustic (LA) modes, respectively.

A typical set of Brillouin spectra from 20% gelatin measured at room temperature is shown in Figure 4. There are two pairs of spectra peaks in each spectrum and a Rayleigh peak at zero frequency. One pair of peaks corresponds to the longitudinal acoustic (LA) mode; the other pair corresponds to the transverse acoustic (TA) mode.

**Figure 5:**
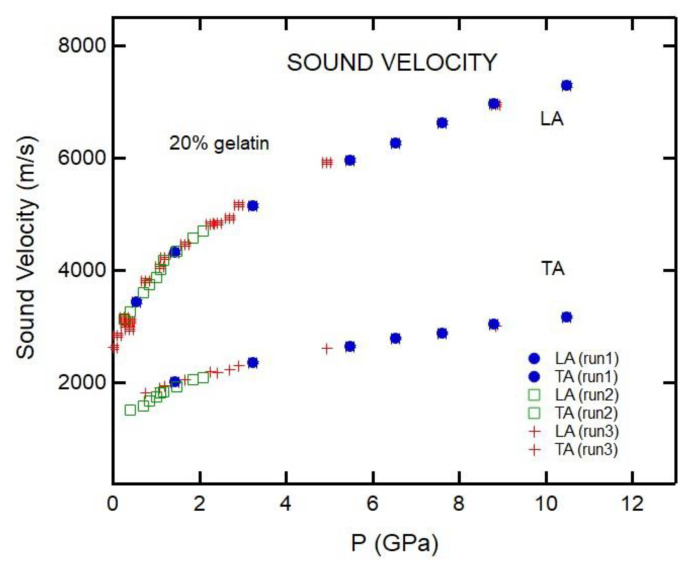
Pressure dependencies of sound velocities of 20% gelatin solutions. The reproducibility was confirmed in preparing samples over different time periods. In each case, the error bars are comparable to the symbol size.

**Figure 6:**
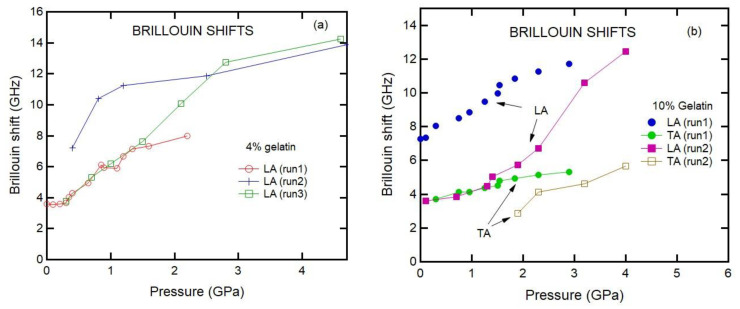
Pressure dependencies of the Brillouin shifts of 4% and 10% gelatin solutions. (**a**) The three experiments were performed for 4% gelatin samples at different time periods; (**b**) two experiments were performed for 10% gelatin solutions.

Figure 5 shows the pressure dependence of sound velocities for the 20% gelatin solutions. The results reveal that pressure dependencies of sound velocities from different experimental runs are consistent. On the other hand, the results for 4% and 10% gelatin differ from run to run (Figure 6). We attribute these differences to sample heterogeneity of the samples as evident from Figure 1.

### 3.2. Density Calculations

Given that the gelatin samples are isotropic, we can calculate the pressure dependence of the density to obtain a pressure–density (pressure–volume, *P*-*V*) equation of state (EOS) from

(3) $$\rho -\rho_{0}=\int_{P_{0}}^{P}\frac{\gamma }{\upsilon_{B}^{2}}dP,$$

where ρ and $\rho_{0}$ are the densities at pressure *P* and *P*_0,_ respectively, $\upsilon_{B}$ is the bulk sound velocity, and $\upsilon_{B}^{2}=\upsilon_{L}^{2}-\frac{4}{3}\upsilon_{T}^{2},\;\mathrm{and}\;\upsilon_{L}$ and $\upsilon_{T}$ are LA and TA velocities, respectively; γ=CPCV ([2,3,5]) is the ratio of heat capacities at constant pressure and constant volume. To fit Equation (3) to the experimental data, we note the definition of the bulk modulus $K=-V\left(\frac{dP}{dV}\right)=\rho \left(\frac{dP}{d\rho }\right)$, which can be measured under isothermal (*K_T_*) or adiabatic (*K_S_*) conditions. The latter is directly related to the bulk sound velocity by $K_{s}=\rho \upsilon_{B}^{2}$, and *K_T_* $=\gamma K_{S}$. Replacing *K_T_* with $\gamma \rho \upsilon_{B}^{2}$, the right side of Equation (3) is integrated to obtain the change in density with pressure. Further, from thermodynamics, we have the following relations between the two heat capacities:

(4) $$C_{P}-C_{V}=VT\frac{\alpha^{2}}{\beta_{T}},\;\mathrm{and}\;\frac{C_{P}}{C_{V}}=\frac{\beta_{T}}{\beta_{S}}=\frac{K_{S}}{K_{T}},$$

where $\alpha =\frac{1}{V}{\left(\frac{\partial V}{\partial T}\right)}_{P}$ is the thermal expansion coefficient, $\beta_{T}=-\frac{1}{V}{\left(\frac{\partial V}{\partial P}\right)}_{T}=\frac{1}{K_{T}}$ is the isothermal compressibility, and $\beta_{S}=-\frac{1}{V}{\left(\frac{\partial V}{\partial P}\right)}_{S}=\frac{1}{K_{S}}$ is the adiabatic compressibility. Thus, the relationship between *K_T_* and *K_S_* is

(5) $$K_{T}-K_{S}=\frac{T\alpha^{2}}{C_{P}}$$

One can estimate the difference between *K_T_* and *K_S_* if the *C_P_* and α are known or vice versa. For example, for crystalline MgO, *K_T_* = 162 GPa and *K_S_* = 160 GPa; thus, γ = 1.01 [24]. For gelatin, the γ is slightly larger than 1 [25]. Given that γ=CPCV≈1 in materials under pressure, we assume a limiting value of unity in the density calculations (Equation (3)).

**Figure 7:**
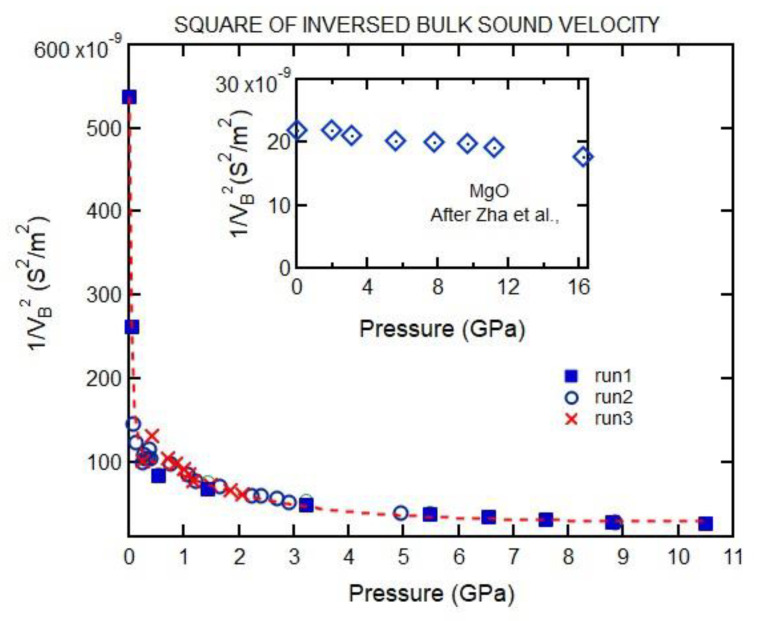
Pressure dependence of the square of inversed bulk velocity; different symbols correspond to the experiments performed at different times. Area under curve gives the density. More data points are obtained at lower pressures to improve the calculation of density. Dashed line is guide for eyes. Inset shows the pressure dependence of the square of inverse bulk sound velocity for crystalline MgO [20].

The density can be determined by integrating the pressure dependence of the square of inversed bulk sound velocity (Equation (3)). Note that the pressure dependence of squared inverse bulk sound velocity shows a drastic change below 1 GPa for gelatin in contrast to, for crystalline materials, the pressure dependence of the square of inversed bulk sound velocity; e.g., see the almost linear behavior for MgO [20] (Figure 7 inset). To reduce the error in the density calculation, we measured the pressure dependence of the sound velocity in small pressure increments below 2 GPa (Figure 7).

**Figure 8:**
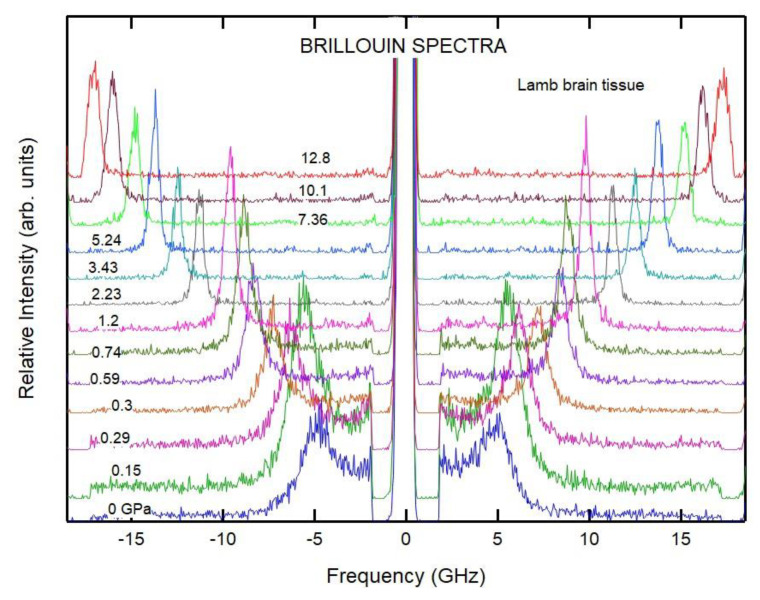
Brillouin spectra of lamb brain tissue at selected pressures. Note that the broad Brillouin peaks at low pressure indicate strong coupling between acoustic modes and concomitant relaxation effects.

**Figure 9:**
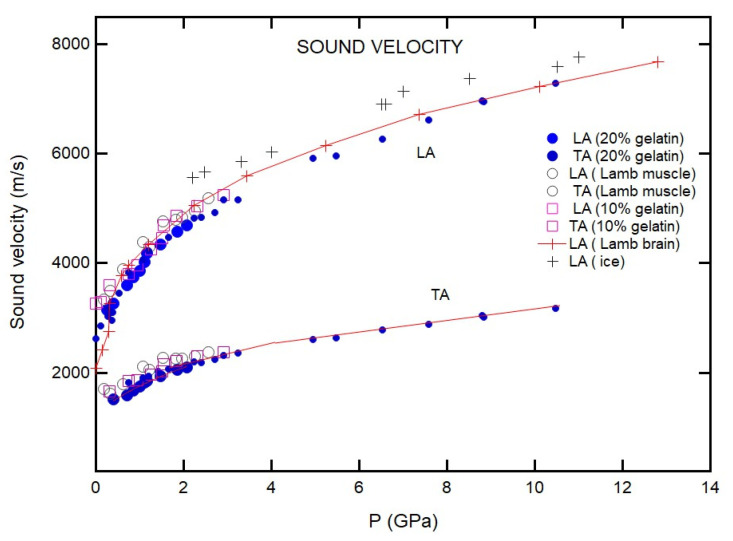
Pressure dependencies of the sound velocities of gelatin solutions, lamb tissues, and H_2_O. Solid circles correspond to the longitudinal and transverse acoustic mode of 20% gelatin sample described in legend; open circles correspond the acoustic modes from lamb muscle tissue, whereas open squares correspond 10% gelatin samples; and crosshair with cross marks correspond to the longitudinal LA mode of the lamb brain tissue. Lines are guides for the eye.

To compare with gelatin, we also measured the pressure dependence of the velocity of lamb muscle and brain tissues. Figure 8 shows the Brillouin spectra from lamb brain tissue at selected pressures. The broad Brillouin peaks at low pressures indicate that the relaxation processes couple strongly with the acoustic modes. The Brillouin shifts from the animal tissue behaved similarly under pressure (Figure 9). Measurements performed on tissue from different animal sources yielded similar results. The TA peaks from the brain tissue sample were found to be extremely weak and were only observed above 4 GPa. On the other hand, the TA peaks were readily observed for the muscle tissue samples (Figure 9).

### 3.3. Equation of State Analysis

The *P*-*V* EOS of gelatin is useful for understanding the behavior of this complex material and can provide information on effective intermolecular interactions. To begin, we analyze the *P*-*V* data with the Tait EOS [26], which was developed for liquids at modest pressures,

(6) $$P=Bexp\left[\frac{1}{C}\left(1-\frac{V}{V_{0}}\right)\right]-B$$

where *V*/*V*_0_ is the relative volume at *P*, and *B* and *C* are empirical parameters. The isothermal bulk modulus at ambient pressure *K*_0_ = *B*/*C* [26]. We also use the Vinet EOS [27]

(7) $$P=3K_{0}\left[\frac{1-{\left(V/V_{0}\right)}^{1/3}}{{\left(V/V_{0}\right)}^{2/3}}\right]exp[\frac{3}{2}\left(K_{0}^{\prime }-1\right)\left(1-\left(V/V_{0}{)}^{1/3}\right)\right]$$

where *K*_0_ is the bulk modulus, *K*_0_′ is its derivative at zero pressure, and *V*_0_ is the initial volume at room temperature.

**Figure 10:**
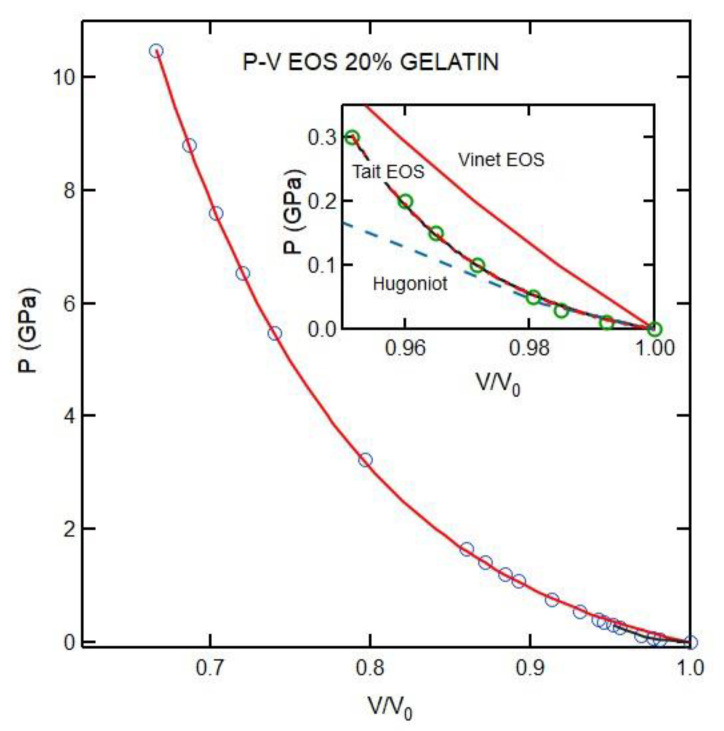
*P*-*V* EOS of 20% gelatin. Open circles are the data obtained from Equation (3). The black and red curves are fits to the Tait and Vinet EOS, respectively. The Tait EOS was fit to data only below 0.5 GPa; a Vinet EOS for data over this range was also performed (see inset); fitting curves are similar for both EOSs. The Tait EOS and Vinet EOS fit to the lower pressure data give similar bulk moduli of 1.7 (±0.2) and 1.6 (±0.2), respectively. Both EOS forms fail to represent the data over the entire pressure range studied. The dashed line is the Hugoniot reported for 20% gelatin [28].

The Tait EOS fit to the data below 0.5 GPa yields a bulk modulus of 1.7 (±0.2) GPa (inset of Figure 10). However, it cannot be used to describe the compression over the entire pressure range or up to 12 GPa. We use Vinet EOS to fit *P*-*V* data up to 12 GPa. We also show that the Vinet EOS fits just the lower pressure range (Figure 10) and obtain a very similar bulk modulus. Given that the Tait EOS was developed for modest compressions (e.g., of fluids) and the Vinet EOS was developed for crystalline solids over a broad range of compression, the results are not surprising. We also include the shock Hugoniot for the 20% gelatin solution [27]. As described in [27], these shock compressions corresponded to extremely low frequency velocity.

### 3.4. Elastic Moduli

**Figure 11:**
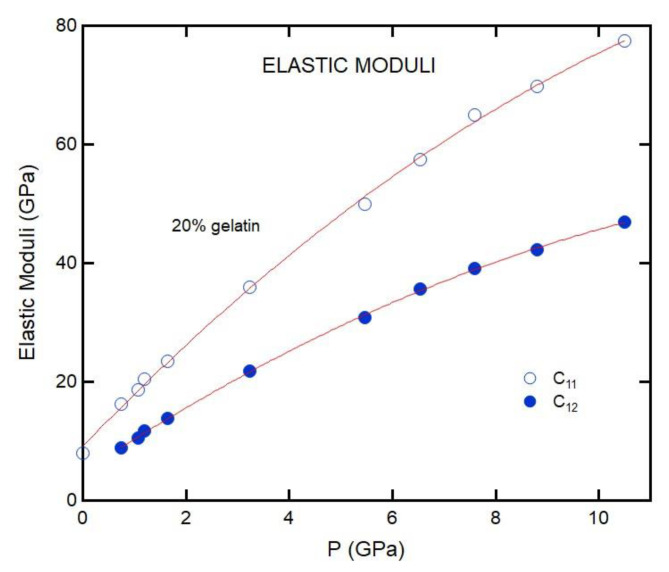
Pressure dependence of the *C*_11_ and *C*_12_ elastic constants for 20% gelatin. The curves are second-order polynomial fit to the data.

**Figure 12:**
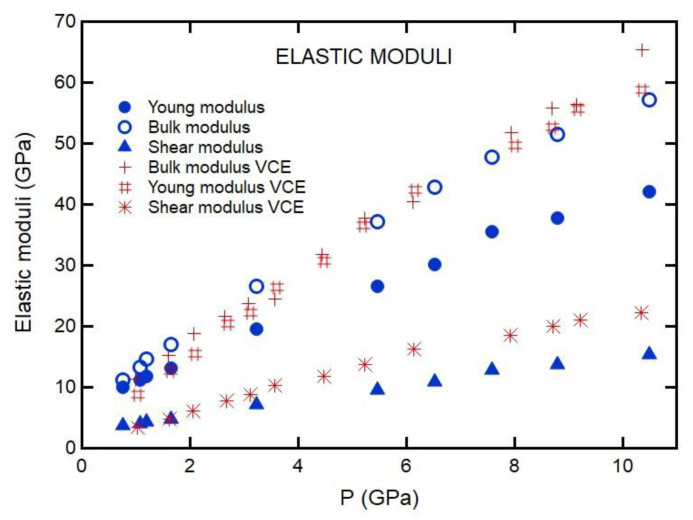
Pressure dependence of bulk, shear, and Young’s moduli.

Elastic moduli *C*_11_ and *C*_12_ (Voigt notation) are directly related to the acoustic velocities through the following:

(8) $$\rho \upsilon_{L}^{2}=C_{11}$$

(9) $$\rho \upsilon_{T}^{2}=\frac{1}{2}\left(C_{11}-C_{12}\right)$$

where *ρ* is the density, *υ_L_* is the LA velocity, and *υ_T_* is the TA velocity. The pressure dependence of *C*_11_ and *C*_12_ for 20% gelatin is shown in Figure 11. We fit the data with the second-order polynomial function to obtain the pressure derivative of the moduli ($\frac{\partial C_{11}}{\partial P}=9.3$ and $\frac{\partial C_{12}}{\partial P}=5.8$ for 20% gelatin). *C*_12_ is approximately half of *C*_11_ for gelatin under pressure. *C*_11_ increases much faster with pressure than *C*_44_ or *C*_12_. Substituted into Equation (9), the ambient pressure transverse TA velocities would be expected to be 1050 m/s for gelatin, corresponding to Brillouin frequency shifts of approximately 2.4 GHz in our measurements. The frequency shifts place the TA peak on the wings of the Rayleigh line, which is, in principle, experimentally resolvable. However, *C*_11_ and *C*_12_ approach one another near ambient pressure (Figure 11), such that in the limit of ambient pressure, the TA velocity goes to zero, collapsing into the Rayleigh line. Furthermore, we compared Young, bulk, and shear moduli obtained for 20% gelatin solution to that of VCE polymer in Figure 12. 

### 3.5. Poisson’s Ratio

Isotropic materials have two independent elastic constants: *C*_11_ and *C*_12_. The pressure dependencies of elastic constants of 20% gelatin are shown in Figure 11. Unfortunately, our lack of knowledge of the initial density of 4% and 10% gelatin and lamb tissue complicates calculating the pressure dependence of density and high-pressure elastic constants. Instead, we can calculate Poisson’s ratio:

(10) $$\sigma =\frac{\upsilon_{L}^{2}-2\upsilon_{T}^{2}}{2(\upsilon_{L}^{2}-\upsilon_{T}^{2})}$$

**Figure 13:**
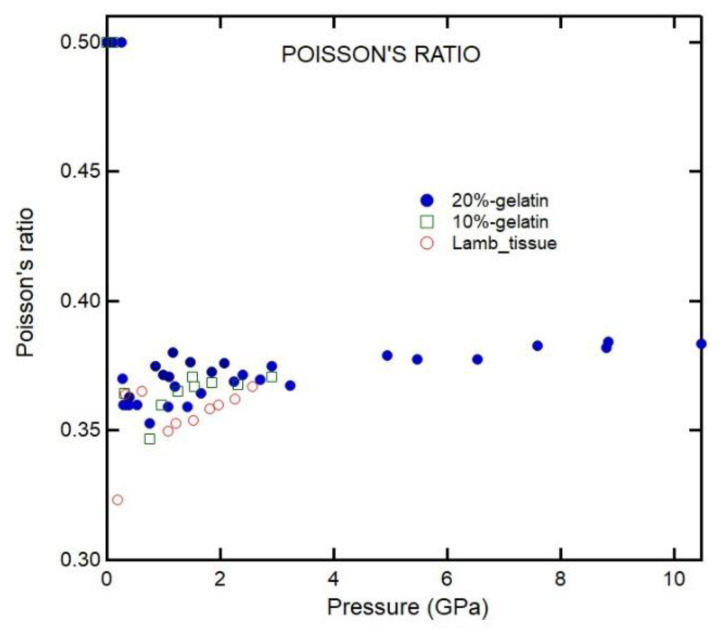
Pressure dependencies of Poisson’s ratio at room temperature. The error in pressure is about 0.2 GPa; given the weakness of TA peaks, Poisson’s ratio has larger relative errors at lower pressures.

The pressure dependencies of Poisson’s ratio *σ* for 10% and 20% gelatin solutions and lamb muscle tissue are shown in Figure 13. The *σ* value for 20% gelatin is 0.36 at 10 GPa, which is comparable with that of silicate glass at the same pressure [16]. The value for a stable, isotropic, and elastic material cannot be less than −1 nor greater than 0.5 because Young’s modulus, the shear modulus, and the bulk modulus must be positive; most materials have a value between 0 and 0.5. Liquid or rubber deformed elastically under small strain has *σ* near 0.5, whereas for metals, the value is around 0.35. For gelatin solutions, particularly at lower pressures, the TA velocities are zero; thus, Poisson’s ratio is 0.5 for that pressure regime. The TA velocities in the Brillouin measurement appear above 0.5 GPa, and *σ* is 0.36, comparable with that of metals and other materials.

### 3.6. Cauchy-Like Relations

The Lame coefficient, *λ*, is given by the following equation:

(11) $$\lambda =K-\frac{2}{3}G$$

where *K* is the bulk modulus and *G* is the shear modulus. For Cauchy’s identity [29], valid for an isotropic substance composed of particles interacting with two-body central forces, the *λ* is identical to *G*, or $K=\frac{5}{3}G$. It is also equal to *M* = 3*G*, where *M* is the longitudinal modulus [29]. The *M*/*G* ratio is an indicator of the character of the force field: if atoms interact through a central potential, *M*/*G* = 3. However, most glasses exhibit behavior suggesting principal effective interactions that are far from those of a central potential or ideal Cauchy relation.

Zwanzig and Mountain [30] generalized the Cauchy-like relation to *M*_∞_ = 3*G*_∞_ + *F*(*T*,*P*) for isotropic liquids, where *M*_∞_ is an unrelaxed longitudinal modulus and *G*_∞_ is an unrelaxed shear modulus, and the term *F*(*T*,*P*) depends on both temperature and pressure. Expanding this argument, Yamura et al. [31] and Krüger et al. [32] found the Cauchy-like relation

(12) $$M_{\infty }=A+BG_{\infty }$$

to hold across the glass transition for many different liquids at ambient pressure, where *A* is a system-dependent parameter and *B* is a parameter found close to 3. Departure from *B* of 3 indicates deviation from a central potential and possible effects of anharmonicity.

**Figure 14:**
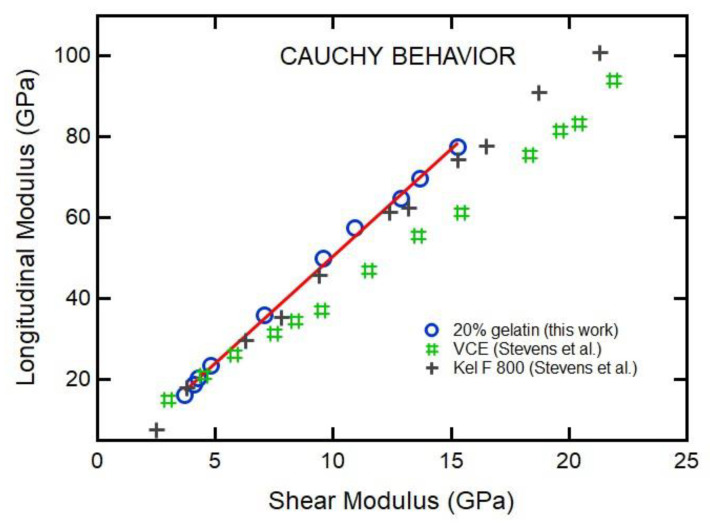
Longitudinal modulus (*M*) as the function of shear modulus (*G*). Open circles represent the data obtained from Brillouin scattering experiments. Linear fit shows a Cauchy-like relationship with the coefficients of *A* = −0.19 ± 0.1 (GPa) and *B* = 5.12 ± 1.3. For comparison, we also listed the data for VCE polymer [9] with *A* = −±1.1 and *B* = 4.2 and Kel-F 800 polymer [11] with *A* = −1.9 ± 1.1 and *B* = 4.9 ± 0.1.

Figure 14 shows the *M* as a function of *G* for various materials. Fitting the data with Equation (12) for 20% gelatin gives *A* = −0.2 (±0.1) and *B* = 5.1 (±1.3). The *B* coefficient is larger than the ideal case: *B* = 3, the original definition of Cauchy relation. Many glass-forming liquids follow a Cauchy-like relation with *B* = 3 at ambient pressure. For example, Scarponi et al. [26] obtained *A* = 1.4 and *B* = 2.8 in a fit of *M*_∞_ = *A* + *BG*_∞_ to data for glycerol across the glass transition temperature at ambient pressure. The closeness of the *B* parameter to 3 suggests that the relevant interaction for glycerol molecules in this regime can be described by a two-body central potential. However, we have found *B* values equal or greater than 4 for many polymers under pressure: VCE polymer has *B* = 4.2 for VCE [6], *B* = 4.9 for Kel-F 800 [7,8], and *B* = 4.4 for amber glass [27]. Thus, *B* = 5.1 for 20% gelatin shows the largest deviation from Cauchy-like behavior, suggesting a strong departure from two-body central interactions within this model.

## 4. Discussion

We first discuss the EOS analysis. As shown in Figure 10 and Table 1, the empirical EOSs we used (Vinet and Tait) do not fit the whole *P*-*V* range well. However, the Vinet EOS fit data between 0.7 and 12 GPa well. On the other hand, the Tait and Vinet EOSs fit well to the data below 0.5 GPa and provide similar bulk moduli for 20% gelatin (Table 1). The inability of a single EOS to fit data over a wide range of compression is also evident for phases of materials that exhibit large pressure stability fields (i.e., ice VII in Table 1). As regards gelatin, we note that the change in the square of inverse bulk velocity with pressure is appreciable (Figure 7). Such a change can be attributed to the collapse of the free volume in polymers on compression. This is consistent with the finding that a single empirical EOS cannot describe the *P*-*V* relation over a wide pressure range (Figure 10). Our results allow us to compare the pressure-dependent elastic moduli of 20% gelatin solution to those of other selected polymers (Figure 12, Figure 13 and Figure 14) [6]. The bulk modulus for 20% gelatin is comparable to that of VCE and sylgard polymers [9]. However, the pressure derivatives of these materials vary considerably (Table 1). In addition, the lamb muscle and brain tissues suggest a lack of shear strength at zero frequency and suggest a strong frequency dependence of velocities and moduli.

The absence of observed shear modes for gelatin at ambient pressure arises from the liquid as opposed to the glassy character of the material. Atomic mobility and even the local structure change across the glass transition, whether determined as a function of temperature (*T*_g_) or pressure (*P*_g_). For example, in PMMA, the pressure dependence of *T*_g_ initially shifts by 200°/GPa, asymptotically approaching a limiting high-pressure value around 0.6 GPa [28]. The ambient pressure *T*_g_ of −73 °C of the gelatin [29] indicates that it would be difficult to observe the shear mode at room temperature and ambient pressure. The shear mode appears above 1 GPa for 20% gelatin at room temperature. Similar behavior is observed for sylgard, with a *T*_g_ of −120 °C and the shear mode observed at 3 GPa by Brillouin scattering. On the other hand, a shear mode is observed for Kel-F 800 polymer at ambient pressure, and its *T*_g_ is near room temperature. A lower *T*_g_ equates to a more flexible polymer backbone and more “liquid-like” behavior.

We point out that, in optical Brillouin scattering, the measured sound wave is in the GHz range. Because of the dependence of the elastomer mechanical properties on frequency, the Brillouin data appear stiffer than the equilibrium measurements regardless of pressure. Indeed, in such complex polymeric systems, the elastic moduli are expected to have a frequency dependence, especially over the pressures measured. Although assessment of this dispersion of the moduli is beyond the scope of this paper, we can make qualitative comments. In glass-forming materials such as gelatin solutions according to the viscoelastic theory [18], the low-frequency-limit velocity (*υ*_0_) approaches the ultrasonic frequencies (Hz–MHz) at high temperature or low pressure with respect to the glass transition temperature *T*_g_ or *P*_g_; when the temperature or pressure approaches the glass transition, the υ_0_ transitions to a high-frequency-limit (GHz) velocity (*υ*_∞_). Such a change in velocity has been observed in glass-forming liquids [18] as a function of temperature [37] or pressure [7,8,21].

LA Brillouin peaks measured at high temperatures [37] and pressures [7,8,21] can be broad, indicating coupling of modes and strong relaxation effects. In high-pressure experiments on fluids, the TA mode is observed when material approaches to the glass transition pressure [7,8]. In the 20% gelatin solution, we observed the TA mode above 0.5 GPa, indicating that the glass transition pressure is near. Therefore, the observed velocity at low pressure is slightly higher than *υ*_0_. This observation is consistent with the shock experiment and our data (Figure 10). Additional studies are needed to address these questions.

## Figures and Tables

**Table 1:** Gelatin and comparison with several polymers and H_2_O. The reference ambient pressure density of 20% gelatin solution used in the analysis is 1.007 gm/cm^3^.

Material	*K*_0_ (GPa)	*K*_0_′	References
20% gelatin	1.7 (±0.2)		This work (Tait < 0.5 GPa)
20% gelatin	1.6 (±0.2)	53 (±3)	This work (Vinet < 0.5 GPa)
20% gelatin	6.1 (±0.2)	7.5 (±0.2)	This work (Vinet)
VCE	2.05	9.99	[9]
estane	2.84	17.1	[9]
sylgard	1.13	8.95	[9]
Kel-F 800	7.5	10	[10]
n-pentane/isopentane	0.45	10.5	[7]
methanol water	0.96 2.2		[33] [34]
ice VII	5 23.7 (±0.9)	8.1 4.15 (±0.07)	[35] [36]

## Data Availability

All original data will be made available on request.
